# Lectotypification of *Tsuga longibracteata* W.C.Cheng (Pinaceae)

**DOI:** 10.3897/phytokeys.172.62450

**Published:** 2021-02-15

**Authors:** Yong Yang, Keith Rushforth

**Affiliations:** 1 College of Biology and Environment, Nanjing Forestry University, 159 Longpan Road, Nanjing 210037, Jiangsu, China Nanjing Forestry University Nanjing China; 2 The Shippen, Ashill, Cullompton, Devon, EN15 3NL, UK The Shippen Devon United Kingdom

**Keywords:** Lectotype, nomenclature, *
Nothotsuga
*, Pinaceae, *Tsuga longibracteata*

## Abstract

W.C.Cheng did not clearly indicate the herbarium repository of the type specimen (*Y.Tsiang 7712*) when he described *Tsuga longibracteata* W.C.Cheng. Later, researchers suggested that the type is either in NAS or in PE. However, we found more than one duplicate of the type collection in both NAS and PE. Following the *Shenzhen Code*, we lectotypify the name *T. longibracteata* with *Y.Tsiang 7712* (PE00003223) that bears a handwritten identification of W.C.Cheng.

## Introduction

Cheng (1932) described an unusual species of *Tsuga* (Endl.) Carrière: *Tsuga longibracteata* W.C.Cheng. This species differs from all known species of *Tsuga* in both vegetative and reproductive characters (Cheng 1932). Its leaves are radially arranged and have stomatal lines on both surfaces; pollen cones are clustered in umbels, and pollen grains possess paired air-bladders; its pedunculate seed cones are more or less erect, and the apical cusp of subspathulate bracts are slightly exserted (Page 1988; Fu et al. 1999; Farjon 2010).

Hu (1951) proposed separating *T. longibracteata* from *Tsuga* and established a new genus “*Nothotsuga*”, but this name was not validly published because Hu did not provide a Latin diagnosis. Page (1988) validated the generic name *Nothotsuga* Hu ex C.N.Page by providing a Latin diagnosis. This segregation has been justified by subsequent molecular systematic studies: *Nothotsuga* diverged from *Tsuga* in the Late Cretaceous (ca. 90 mya, Havill et al. 2008). *Nothotsuga* is now recognized as a distinct genus, with the only extant species being distributed in southern China including Fujian, Guangdong, Guangxi, Guizhou, Hunan, Jiangxi, and Yunnan (Fu et al. 1999; Farjon 2010).

Cheng (1932) designated *Y.Tsiang 7712* as the type of the species name *Tsuga longibracteata*, but did not clearly indicate which specimen is the holotype or where the type specimen is deposited. We found 12 specimens deposited in eight international herbaria, i.e. three specimens in PE (PE00003225, PE00003224 and PE00003223), two in NAS (NAS00070064 and NAS00070063), two in HUH (A00052508 and A00052510), one each in E (E00215871), IBSC (IBSC0012857), K (K000288277), NY (NY00001279), and S (S-C-4796) respectively. Farjon (2010) indicated that the holotype is in NAS, but Lin (2014) recorded the specimen in PE (PE00003223) as the holotype. Under Art. 7.11 of the *Shenzhen Code* (Turland et al. 2018), neither designation can be considered an effective lectotypification because their books were published after 1 January 2001 and did not include the phrase “designated here” (hic designatus) or an equivalent. The two specimens in NAS are poorly preserved and have printed labels without any handwriting. However, one of the two specimens in PE (PE00003223) bears Cheng’s handwritten identification and is relatively well preserved. Accordingly, we choose to lectotypify *Tsuga longibracteata* with this specimen.

## Typification

### Tsuga longibracteata

Taxon classificationPlantaePinalesPinaceae

W.C.Cheng, Contrib. Biol. Lab. Sc. Soc. China, Bot. Ser. vii. 1 (1932).

1A55C046-BD56-5079-A68C-4A134EA9F396

Fig. 1.

≡Nothotsuga longibracteata (W.C.Cheng) Hu ex C.N.Page, Notes Roy. Bot. Gard. Edinburgh 45(2): 390 (1988, published in 1989).

#### Type.

China. Guizhou (贵州): Yinjiang Tujiazu Miaozu Zizhixian (印江土家族苗族自治县, as “Yin-Kiang”), Fanjing Shan (梵净山, as “van-ching-shan”), in densely shaded ravine, alt. 400–500 m, 19 December 1930, *Y.Tsiang* (蒋英) *7712* (Lectotype: PE00003223, designated here; isolectotypes: A00052508, A00052510, E00215871, IBSC0012857, K000288277, NAS00070063, NAS00070064, NY00001279, PE00003225, PE00003224, S-C-4796).

**Figure 1.**
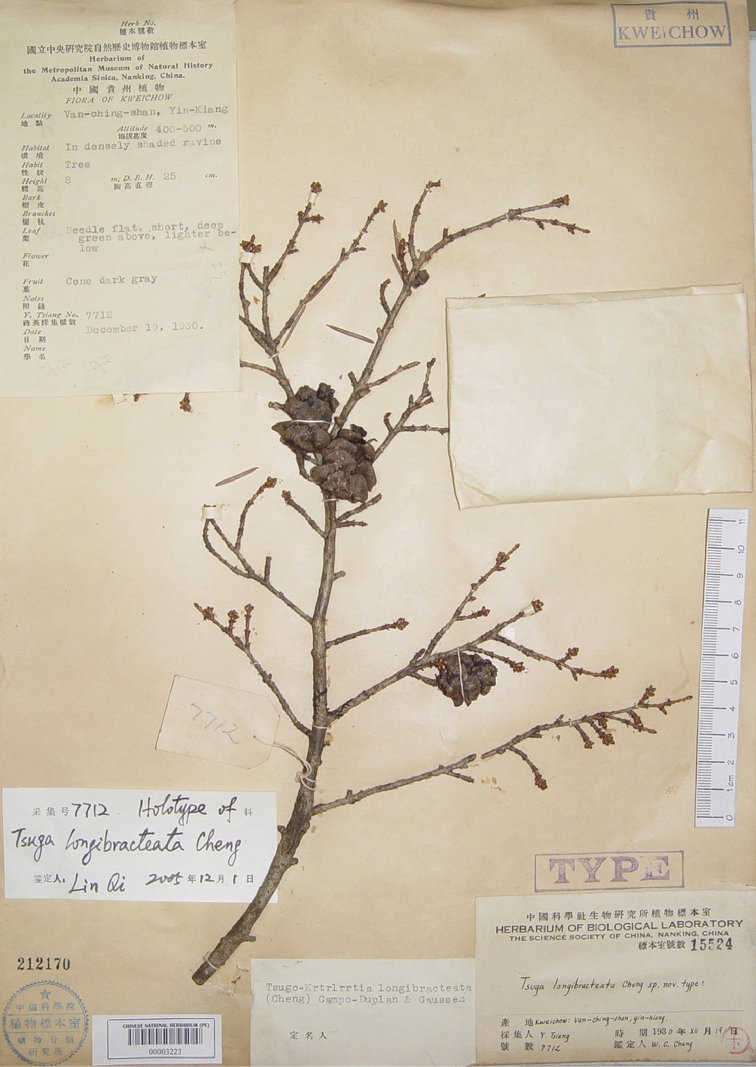
Lectotype of *Tsuga longibracteata* W.C. Cheng [≡*Nothotsuga longibracteata* (W.C.Cheng) Hu ex C.N.Page]: *Y.Tsiang* (蒋英) *7712* (PE00003223).

## Supplementary Material

XML Treatment for Tsuga longibracteata
